# Autophagy Upregulation by the TFEB Inducer Trehalose Protects against Oxidative Damage and Cell Death Associated with NRF2 Inhibition in Human RPE Cells

**DOI:** 10.1155/2020/5296341

**Published:** 2020-07-21

**Authors:** Samuel Abokyi, Sze wan Shan, Chi-ho To, Henry Ho-lung Chan, Dennis Yan-yin Tse

**Affiliations:** ^1^School of Optometry, The Hong Kong Polytechnic University, Hong Kong SAR, China; ^2^Department of Optometry & Vision Science, University of Cape Coast, Cape Coast, Ghana

## Abstract

Trehalose is a natural dietary molecule that has shown antiaging and neuroprotective effects in several animal models of neurodegenerative diseases. The role of trehalose in the management of age-related macular degeneration (AMD) is yet to be investigated and whether trehalose could be a remedy for the treatment of diseases linked to oxidative stress and NRF2 dysregulation. Here, we showed that incubation of human retinal pigment epithelial (RPE) cells with trehalose enhanced the mRNA and protein expressions of TFEB, autophagy genes *ATG5* and *ATG7*, as well as protein expressions of macroautophagy markers, LC3B and p62/SQTM1, and the chaperone-mediated autophagy (CMA) receptor LAMP2. Cathepsin D, a hydrolytic lysosomal enzyme, was also increased by trehalose, indicating higher proteolytic activity. Moreover, trehalose upregulated autophagy flux evident by an increase in the endogenous LC3B level, and accumulation of GFP-LC3B puncta and free GFP fragments in GFP-LC3 – expressing cells in the presence of chloroquine. In addition, the mRNA levels of key molecular targets implicated in RPE damage and AMD, such as vascular endothelial growth factor- (*VEGF*-) A and heat shock protein 27 (HSP27), were downregulated, whereas *NRF2* was upregulated by trehalose. Subsequently, we mimicked *in vitro* AMD conditions using hydroquinone (HQ) as the oxidative insult on RPE cells and evaluated the cytoprotective effect of trehalose compared to vehicle treatment. HQ depleted NRF2, increased oxidative stress, and reduced the viability of cells, while trehalose pretreatment protected against HQ-induced toxicity. The cytoprotection by trehalose was dependent on autophagy but not NRF2 activation, since autophagy inhibition by shRNA knockdown of *ATG5* led to a loss of the protective effect. The results support the transcriptional upregulation of TFEB and autophagy by trehalose and its protection against HQ-induced oxidative damage in RPE cells. Further investigation is, therefore, warranted into the therapeutic value of trehalose in alleviating AMD and retinal diseases associated with impaired NRF2 antioxidant defense.

## 1. Introduction

The etiology of age-related macular degeneration (AMD) is multifactorial and includes both genetic and environmental risk factors [1, 2]. Oxidative damage to the retinal pigment epithelium (RPE), however, appears to play a crucial role based on *in vivo* studies in AMD subjects and animal models of retinal degeneration, as well as *in vitro* cell culture models of AMD [3]. The increased risk of developing AMD among cigarette smokers and the intimate relationship between the number of pack-years of smoking and disease progression are compelling evidence implicating oxidative stress in AMD [4, 5]. Experimental studies further our understanding of the association between smoking and AMD by demonstrating that the RPE is susceptible to oxidative damage upon exposure to cigarette smoke or its prooxidant hydroquinone (HQ) [6]. In mice, it was found that prolonged exposure to cigarette smoke damaged the RPE and led to AMD-like retinal changes [6]. These findings elucidate the primary role of oxidative RPE damage in the development of AMD.

Nuclear factor erythroid 2-related factor 2 (NRF2) activation is a master antioxidant transcription factor that regulates oxidative stress [7, 8]. Under normal basal conditions, the NRF2 antioxidant transcription factor is bound to the Kelch-like ECH-associated protein 1 (KEAP1) in the cytosol, and its level is tightly regulated via the ubiquitin-proteasome system (UPS) [7]. The activation of NRF2 occurs when it disassociates from the KEAP1 repressor. Consequently, NRF2 stabilizes and translocates into the nucleus leading to the activation of the antioxidant response elements (ARE) for the induction of detoxifying (phase II enzymes) and antioxidant enzymes [9]. Hence, NRF2 activation under oxidative stress protects against oxidative damage and promotes cell survival. However, postmortems conducted on specimens from eyes with AMD have shown that the NRF2 antioxidant transcription factor was downregulated in RPE cells overlying drusen [10]. Studies have reported that the major risk factors of AMD, including aging and cigarette smoking, promote oxidative damage to RPE cells by inhibition of NRF2 antioxidant defense [11, 12]. In aged rats, there is the inhibition of NRF2 mRNA, reduced antioxidant enzymes, and increased oxidative stress in the RPE, promoting NaIO_3_-induced retinal degeneration [12]. We also observed that HQ depleted NRF2 and increased oxidative damage to RPE cells *in vitro*, consistent with the effect of cigarette smoking on primary bronchial epithelial cells of patients with chronic obstructive pulmonary disease (COPD) [13]. Hence, we proposed that since oxidative damage of the RPE and AMD was associated with NRF2 inhibition, targeting an alternative robust antioxidant pathway could be an effective approach to protect against RPE damage and AMD.

Autophagy is a catabolic mechanism involving lysosomal degradation of cytosolic material, including macromolecules and organelles. In mammals, three autophagic pathways target substrates for lysosomal degradation [14]. Macroautophagy involves the formation of autophagosomes, double-membrane vesicles, to transport substrates to lysosomes for degradation. In chaperone-mediated autophagy (CMA), lysosomal degradation is regulated by the interaction between lysosome-associated membrane protein 2 (LAMP2), a lysosomal receptor, and the chaperone Hsc70, facilitating the selective access of substrates with the target motif (KFERQ) into the lysosome. Lysosomal degradation by microautophagy is the simplest, involving the direct invagination and sequestration of substrates into the lysosome [14]. Studies of neurodegenerative diseases linked to oxidative stress, such as Alzheimer's, Parkinson's, Huntington's disease, amyloid lateral sclerosis, and AMD, revealed that autophagy induction conferred cytoprotection [15, 16]. Based on the emerging role of autophagy in neurodegeneration, the inducers of transcription factor EB (TFEB), a major regulator of autophagy and lysosomal biogenesis, have received considerable attention [17, 18]. TFEB coordinates multiple steps in the autophagy-lysosomal pathway via the activation of the coordinated lysosomal expression and regulation (CLEAR) network of genes [19].

Recently, a small molecule called trehalose, a naturally existing disaccharide of known neuroprotection against numerous neurodegenerative disease models [20, 21], was found to be a potent TFEB inducer in neurons [22, 23]. Trehalose is safe and approved as a food ingredient for human consumption by the European regulatory system and the U.S. Food and Drug Administration in 2000 [24]. This disaccharide is in use for topical and systemic treatment of ocular and systemic disorders, including oculopharyngeal muscular dystrophy, spinocerebellar atrophy type 3, atherosclerosis, fatty liver disease, dry eyes, and in the postsurgical management of laser-assisted in situ keratomileusis (LASIK) in multiple clinical trials [25–28],(https://clinicaltrials.gov/ct2/show/NCT03700424; https://clinicaltrials.gov/ct2/show/NCT03738358). While autophagy upregulation by trehalose is well reported in many studies [23, 29], a few reports have disputed this claim [30, 31]. In this study, autophagy induction by trehalose on human RPE cells was investigated. Additionally, its cytoprotective role against hydroquinone, an oxidant that impairs the NRF2 transcription factor, was explored. The results support trehalose as a unique autophagy inducer in human RPE cells. The disaccharide upregulated autophagy flux and the mRNA or protein levels of key autophagy regulators, including TFEB, ATG5 and ATG7, LC3B, LAMP2, and the lysosomal hydrolase cathepsin D. Collectively, the evidence supports an upregulation of crucial stages in the autophagy-lysosomal pathway, such as (1) the initiation of autophagosome (2) autophagosome formation and (3) autophagosome maturation, autolysosome formation, and substrate degradation. Moreover, the potential therapeutic benefit of trehalose in AMD was ascertained by demonstrating its autophagy-dependent cytoprotection against the cigarette smoke oxidant HQ in human RPE cells.

## 2. Materials and Methods

### 2.1. Culture of ARPE-19 Cells

Human RPE cells (ARPE-19, ATCC® CRL2302 ™) were cultured in Dulbecco's modified Eagle's medium (DMEM)/F12 (Sigma-Aldrich, St. Louis, MO, USA) containing 10% fetal bovine serum (Invitrogen-Gibco, Grand Island, NY, USA) and 1% penicillin-streptomycin antibiotic mixture (Gibco™ 10,000 UmL^−1^, Thermo Fisher Scientific). The cell line was thoroughly tested for mycoplasma using three different methods—agar culture (direct) method, Hoechst DNA stain (indirect) method, and PCR assay (lot #: 70022669, ATCC®). Cells were incubated at 37°C in a humidified atmosphere of 5% carbon dioxide and the medium changed every three days until the cells reached 90% confluency for use in experiments. Passages 16-25 were used for all experiments.

### 2.2. Cell Viability Assay

Trypan blue dye exclusion assay was used to assess cell viability. In brief, 1x10^6^ cells/well into 6-well plates and incubated until 90% confluency was achieved. Cells were then incubated with the appropriate dose of trehalose (Sigma-Aldrich, T9449), 50 *μ*M HQ (hydroquinone, Sigma-Aldrich, H9003), vehicle, or a combination of these drugs as desired. Each treatment was performed in triplicate. Cells were then detached by trypsin and stained with trypan blue for viability and total and viable cells enumerated to determine percentage viability.

### 2.3. CM-H2DCFDA Assay for Intracellular Reactive Oxygen Species (ROS)

Cells (5x10^4^ cells/well) were transferred into 96-well plates for incubating overnight, followed by incubation with 0-100 mM trehalose in serum-free medium for 24 h. The medium was then removed, the cells washed with PBS, and incubated with 5 *μ*M of 5-(and-6)-chloromethyl-2′,7′-dichlorodihydrofluorescein diacetate (CM-H_2_DCFDA, C6827, Invitrogen) in the dark at 37°C for 1 h. On uptake by the cells, the probe is oxidized by intracellular ROS, converting it from its nonfluorescent state to a fluorescent form. Fluorescence intensity was measured at 483 nm against 530 nm as reference using a Clariostar microplate reader (BMG Labtech, Offenburg, Germany).

### 2.4. Determination of Protein Carbonyl Level by ELISA

Protein carbonyl concentration was measured in protein samples using the Oxiselect™ protein carbonyl ELISA kit (Cell Biolabs, STA-310) according to the manufacturer's instructions. Briefly, the protein samples prepared from cells preincubated with trehalose or vehicle before exposure to HQ were incubated with 1% streptomycin sulfate (Sigma-Aldrich, S9137) and diluted at the specified 10 *μ*g/ml protein concentration. Protein samples were then adsorbed on a 96-well plate for 2 h at 37°C and derivatized to dinitrophenylhydrazine (DNPH). After treating with anti-DNP antibody and followed by Horseradish Peroxidase (HRP) conjugated secondary antibody, the absorbance was measured at 450 nm wavelength using a microplate reader (Ao, Azure Biosystems Inc., Dublin, USA).

### 2.5. Proteasome Activity Assay

Proteasome activity was measured using a fluorogenic 7-amino-4-methyl coumarin- (AMC-) tagged substrate kit to detect chymotrypsin-like activity, following the manufacturer's protocol (Biovision proteasome activity assay kit, San Francisco, CA). Briefly, ARPE-19 cells incubated with trehalose or vehicle for 24 h were lysed with 25 mM Tris-HCl buffer for 60 min at 4°C, and the supernatant collected by centrifugation at 13,000 rpm for 10 min at 4°C. The cell lysates of samples were loaded onto a 96-well plate in duplicate for incubation, either with the fluorescent substrate at 37°C for 30 min in the presence of MG132 (proteasome inhibitor) or without (as control). The proteasome has chymotrypsin-like which releases free, highly fluorescent AMC from the AMC-tagged peptide substrate. Fluorescence intensity was then measured at 350 nm against 440 nm as a reference using a Clariostar microplate reader (BMG Labtech, Offenburg, Germany). Protein concentration was used for the normalization of the data.

### 2.6. shRNA Knockdown of ATG5

Stable knockdown of *ATG5* in ARPE-19 cells was performed using lentivirus to deliver short hairpin RNA (shRNA). An aliquot of 3 x 10^6^ cells of HEK293T cells were seeded into 10 cm culture dishes. A scramble shRNA-coding lentiviral vector (Addgene plasmid # 1864) was used to transfect the cells with lentiviral particles with either scrambled shRNA plasmid or ATG5 shRNA, TRC numbers: TRCN0000151474 (Sigma Alrich) using Lipofectamine 2000 (Invitrogen) following the manufacturer's instructions. After 8 hours, the medium was changed and incubation continued for another 48 to 52 hours. Virions were collected and precipitated overnight with polyethyleneglycol (PEG) before filtering through a 0.45 *μ*m filter and finally transduced into ARPE-19 cells for 48 hours. The cells were then subjected to puromycin (1.0 *μ*g/mL) selection for 10 days for the identification of resistant colonies.

### 2.7. Flow Cytometry for Apoptosis

Cellular apoptosis was investigated using the FITC Annexin V/PI kit and following the recommended protocol (BioLegend Inc., San Diego, USA). Briefly, 1 × 10^6^ cells from different treatments were trypsinized, rinsed twice with cold cell staining Buffer, and suspended in 100 *μ*l of binding buffer. Subsequently, 5 *μ*l Annexin V-FITC was added and incubated for 10 min in dark condition at RT, followed by the addition of 10 *μ*l PI for 5 min before flow cytometry (BD FACSVia Flow Cytometer, BD Biosciences, USA).

### 2.8. Autophagy Flux Assessment by GFP-LC3 Puncta and GFP-LC3 Cleavage Assay

Cells expressing GFP-LC3 were generated by transfection of ARPE-19 cells grown in 6 well-plates and reaching 80% confluency with 2.5 *μ*g pEGFP-LC3 plasmid (Addgene plasmid # 24920) using Lipofectamine 3000 (Invitrogen) for 24 h. After the desired treatments, there was autophagy flux assessment by immunoblotting for free GFP levels in a cell lysate or live-fluorescence cell imaging for GFP-LC3 puncta in the cells. For quantification of GFP-LC3 puncta, treated cells were rinsed with PBS and incubated with serum-free medium for fluorescence imaging using an inverted confocal microscope (Eclipse Ti2-E, Nikon Instruments Europe B.V., Amsterdam) and a 40X objective. GFP-LC3 puncta were quantified from triplicates by counting a total of 30 cells as previously reported [32].

### 2.9. Treatment, Protein Extraction, and Immunoblotting

After treatment of cells with trehalose, HQ (hydroquinone, Sigma-Aldrich, H9003), MG132, CQ (Chloroquine diphosphate salt, C6628, Sigma-Aldrich), NH4Cl (A9434, Sigma-Aldrich), vehicle, or a combination of these as designated, cells were trypsinized (0.25% trypsin), pelleted, and washed twice with PBS before protein or RNA extraction. Proteins were extracted using ice-cold 1X RIPA lysis buffer [0.5 M Tris-HCl (pH 7.4), 1.5 M NaCl, 2.5% deoxycholic acid, 10% NP-40, and 10 mM EDTA (Millipore, Temecula, CA)] supplemented with protease and phosphatase inhibitor tablets (Roche Applied Science, Indianapolis, IN). Samples were sonicated for 1 h in ice at 4°C, followed by centrifugation at 18,000 g for 30 min at 4, °C and collection of the supernatant. Bradford assay was performed to determine the protein concentration of samples and the absorbance read at 595 nm wavelength on a microplate reader (Ao, Azure Biosystems Inc., Dublin, USA).

An equal amount of denatured proteins (total protein of 30 *μ*g) of the samples was loaded in a gel for SDS-PAGE electrophoresis (10% SDS-PAGE gel). Proteins were electrotransferred from the gel to an Immobilon-FL PVDF membrane (Millipore) for 2 h at 250 mA. The membrane was then blocked using 5% nonfat milk in Tris-buffered saline containing 0.05% Tween 20 (Bio-Rad Laboratories) for 1 h at room temperature. Primary antibody incubation with anti-TFEB (D2O7D, Cell Signaling Technology, 1 : 500), anti-LC3 (NB100-2220, Novus Biologicals, dilution 1 : 1000), anti-p62 (2C11, Novus Biologicals, dilution 1 : 2000), anti-NRF2 (EP1808Y, Abcam, dilution 1 : 1000), anti-LAMP2 (sc-18822, Santa Cruz Biotechnology, dilution 1 : 2000), anti-cathepsin D (sc-377299, Santa Cruz Biotechnology, dilution 1 : 500), anti-HSC 70 (sc-7298, Santa Cruz Biotechnology, dilution 1 : 1000), or anti-GFP (sc-9996, Santa Cruz Biotechnology, dilution 1 : 1000) was performed. The membrane was washed three times followed by incubation with HRP-conjugated secondary antibodies (anti-mouse IgG (H+L), A16066, or anti-rabbit IgG (H+L), A16110; dilution 1 : 2000) (Thermo Fisher Scientific). After washing, a mixed enhanced luminol-based chemiluminescent (ECL) substrate solution was incubated with the membrane, and the immunoreactive bands imaged using the Azure c600 imaging system. (Azure Biosystems; Dublin, CA). Quantification of bands was performed using ImageJ analysis software. Protein expressions were normalized to GAPDH (AM4300, anti-GAPDH, dilution 1 : 2000, Thermo Fisher).

### 2.10. Isolation of RNA, RT-PCR, and qPCR

Briefly, RNA extraction was performed using Trizol (Invitrogen, Carlsbad, CA, USA) following the manufacturer's suggested protocol. cDNA was transcribed from 1 *μ*g total RNA using the High Capacity cDNA Reverse Transcription Kit (Applied Biosystems; Thermo Fisher Scientific, Inc., Waltham, MA, USA) and stored for conventional reverse transcription-polymerase chain reaction (RT-PCR) or real-time quantitative PCR (qPCR).

Quantitative PCR was performed in triplicate using a reaction mix of 2 *μ*l cDNA template, 5 *μ*l LightCycler 480 SYBR Green I Master mix (Roche Diagnostics), 1 *μ*l nuclease-free water, and 1 *μ*l of gene-specific primers (Table 1). Following denaturation at 95°C for 5 min, 40 cycles at 95°C for 30 s, 60°C for 30 s, and 72°C for 30 s were run using the LightCycler®480 Instrument II (Roche Diagnostics, Mannheim, Germany). Fold changes were calculated using the change in the Cycle threshold (*∆∆*CT) method.

For conventional RT-PCR, the 20 *μ*l reaction mix contained 1.5 *μ*l cDNA template, 10 *μ*l 2x Taq HS mix (R028A, Premix Taq™ DNA Polymerase Hot-Start Version), 1 *μ*l each of forward and reverse primers (10 *μ*M) and 6.5 *μ*l nuclease-free water. Amplification was performed for 25 cycles of 94°C for 30 s, 60°C for 30 s, and 72°C for 30 s (MJ Research PTC-200 Gradient Thermal Cycler, USA). The amplified products were loaded and analyzed by 1.5% agarose gel electrophoresis containing GelGreen nucleic acid stain (Biotium Inc., Hayward, CA, USA) and visualized under UV light (Gel Doc/ChemiDoc Imager, Azure, Dublin, CA, USA). B-actin was used as a reference for the normalization of expression of other genes.

### 2.11. Data Analysis

Data were analyzed using GraphPad Prism. All data are presented as mean ± SD. Unpaired *t*-test or One-way ANOVA followed by multiple comparison tests was used as appropriate to determine differences between treatments. Statistical significance was set at *p* < 0.05.

## 3. Results

### 3.1. Trehalose Increased Autophagy Flux in RPE Cells

**Figure 1:**
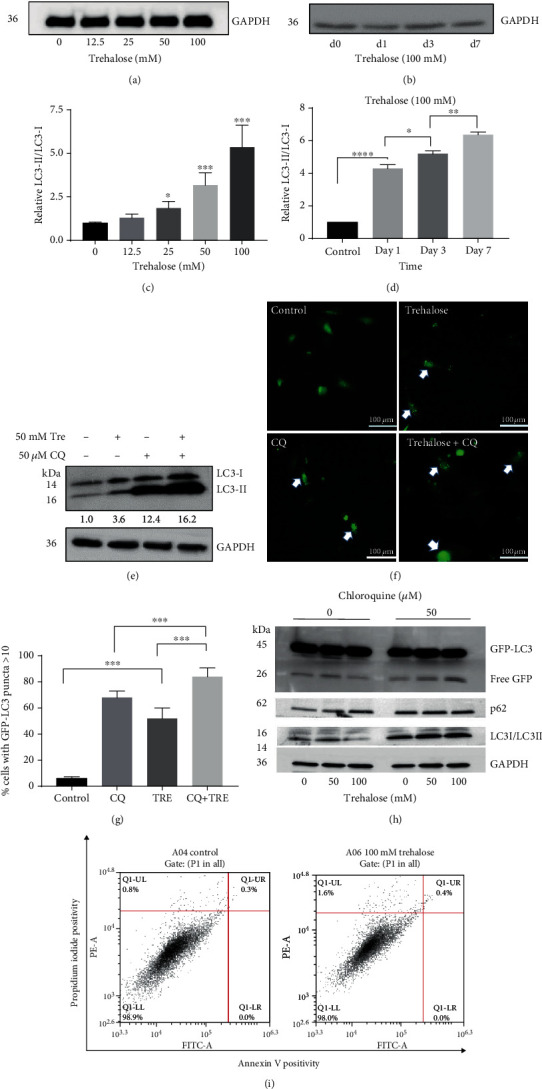
Trehalose increased autophagosomes formation and autophagy flux. (a–e) Endogenous LC3II expression in cell lysate from ARPE-19 cells treated as designated. (f, g) Live-fluorescence microscopy showing percentage of GFP-LC3–expressing cells containing >10 GFP-LC3 puncta after treatment. (h) Free GFP levels in cell lysate from GFP-LC3–expressing cells after treatment. (i) Flow cytometry results of Annexin V/PI staining in wildtype cells after trehalose treatment. Percentage of late apoptosis and early apoptosis are shown in the upper right and lower right quadrants, respectively. Data represent the mean ± SD of 3 independent experiments. Statistical analysis was performed by one-way ANOVA followed by multiple comparison tests. ^∗^*p* < 0.05, ^∗∗^*p* < 0.01, ^∗∗∗^*p* < 0.001.

Autophagosome formation is essential in autophagy degradation [33]. Due to the relevance of autophagosome cargoes in autophagy, its monitoring using the lipidated LC3 (LC3-II), an autophagosome membrane-bound protein, provides vital information about the process. Without any obstruction of the autophagy flux, the accumulation of LC3-II correlates with the induction of autophagy [34]. Hence, to determine whether trehalose induced autophagy, the changes in LC3-II protein expression levels were evaluated. Incubating ARPE-19 cells with varying doses of trehalose led to the accumulation of LC3-II dose-dependently (Figures 1(a) and 1(c)), indicating an increase in autophagosomes by trehalose. Additionally, the expression of LC3-II increased time-dependently when cells were incubated with 100 mM trehalose (Figures 1(b) and 1(d)).

Next, changes in the autophagy flux by trehalose in the presence of the autophagy inhibitor chloroquine (CQ) were investigated, by assessment of endogenous LC3-II levels, the accumulation of GFP-LC3 puncta in GFP-LC3 transfected cells, and the formation of free GFP fragments due to the proteolytic cleavage of GFP-LC3. To inhibit autophagy, cells were incubated with 50 *μ*M CQ, due to the toxicity and dose-response changes previously reported ([35, 36]). In the presence of CQ, trehalose treatment increased the endogenous LC3-II expression, indicating an upregulation of autophagy flux by trehalose (Figure 1(e)). Moreover, the diffuse cytosolic staining pattern of the GFP-LC3-expressing cells became more punctate with trehalose treatment (Figure 1(f)), reflecting higher recruitment of GFP-LC3 to autophagosome membranes [37]. The data showed that the percentage of cells with GFP − LC3 > 10 puncta was increased by trehalose (*p* < 0.001, Figure 1(g)).

Furthermore, analysis of the proteolytic degradation of GFP-LC3 in the transfected cells by trehalose also confirmed the upregulation of autophagy flux. There was a dose-dependent increase in free GFP level by trehalose in the presence of CQ (Figure 1(h)), revealing elevation in the degradation of GFP-LC3 within autolysosomes since the LC3 portion of the fusion protein is rapidly degraded than GFP [32]. Without CQ, however, free GFP level was increased when cells were treated with 50 mM trehalose but decreased with a higher trehalose dose (100 mM) (Figure 1(h)). When the lysosomal activity is very high, both free GFP fragments and LC3 portions are degraded together, as happens under prolonged starvation [32]. This may explain why there was a decline in free GFP levels with 100 mM trehalose treatment in the absence of CQ, but in the presence of CQ, this was reversed because CQ impairs lysosomal degradation revealing the true level of autophagy flux [32]. Collectively, the results corroborate increased autophagosome formation and autolysosome degradation by trehalose in RPE cells.

### 3.2. Autophagy Induction by Trehalose Is Not Dependent on Apoptosis

Cells with phagocytic functions, including RPE, liver cells, and macrophages, respond to apoptosis by inducing LC3B-associated phagocytosis (LAP) to restore cellular homeostasis [38, 39]. There is, therefore, the possibility that trehalose promotes apoptosis, causing the induction of LAP, which is being confused with canonical autophagy [39, 40]. Additionally, LC3B accumulation due to the upregulation of autophagy could be a transduction signal for apoptotic cell death [41]. Hence, it was investigated whether trehalose-induced LC3B was linked to apoptosis, by studying the annexin V/PI staining pattern of trehalose-treated cells using flow cytometry. It was found that 100 mM concentration of trehalose did not alter the apoptotic pathway (Figure 1(i)), elucidating that autophagy induction by trehalose was not dependent on apoptosis and did not promote apoptotic cell death.

### 3.3. Trehalose Upregulates p62 mRNA and Protein Expression Levels and p62 Turnover

**Figure 2:**
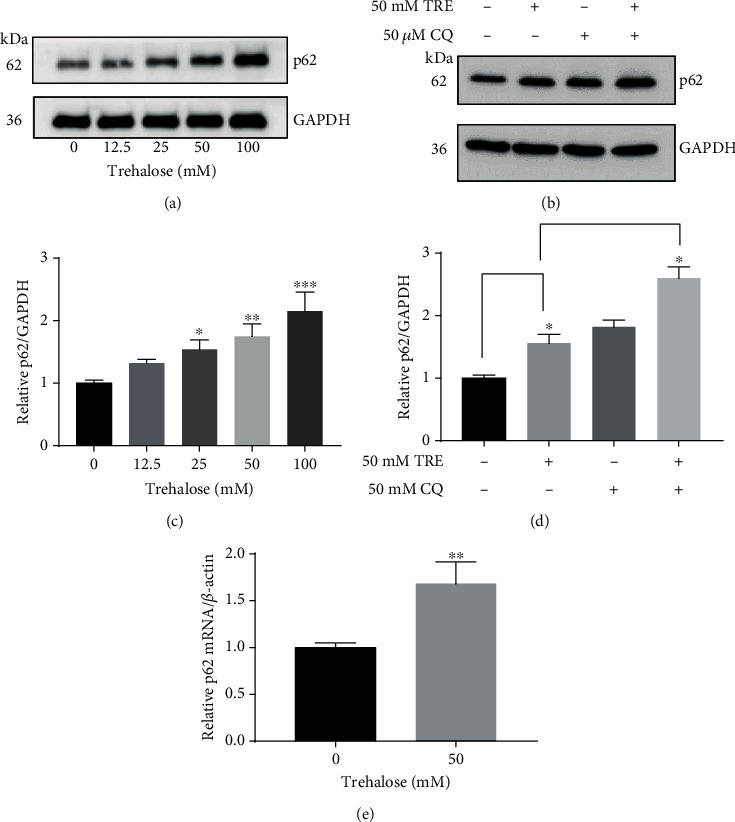
Effect of trehalose on p62 mRNA and protein expression levels and p62 turnover. (a, e) Trehalose upregulated p62 expression at the transcription and protein levels. (a–d) Immunoblotting with p62 antibodies in whole-cell lysate from treated cells. (e) RNA extraction for RT-qPCR to evaluate the gene expression level of p62. (b) Trehalose increased p62 in the presence of chloroquine (CQ). Data represent the mean ± SD of 3 independent experiments. Statistical analysis was performed by the unpaired *t* test or one-way ANOVA followed by multiple comparison tests. ^∗^*p* < 0.05, ^∗∗^*p* < 0.01, ^∗∗∗^*p* < 0.001 vs. control.

The p62/SQSTM protein level may change in cells during autophagy induction [42]. This is because the p62 adaptor protein binds to other cytosolic autophagy substrates and becomes sequestered by autophagosomes, through interaction with LC3-II, which is degraded by lysosomes [43]. Hence, the p62 protein level is reduced when autophagy is stimulated [44]. It was observed that trehalose increased p62 protein expression dose-dependently in the RPE cells (Figures 2(a) and 2(c)). It was investigated whether the elevated p62 protein expression level in the cells incubated with trehalose was related to transcriptional upregulation of p62, as occurs in prolonged starvation-induced autophagy [45]. The 50 mM trehalose was subsequently used when cotreatment of cells with trehalose, and other drugs was desired because it had been determined that it was the safest and optimal dose that did not cause any changes in cell viability and morphology. Quantitative PCR confirmed the upregulation of p62 mRNA expression in cells incubated with 50 mM trehalose compared to control (Figure 2(e)). Thus, the accumulation of p62 in cells treated with trehalose may be related to the upregulation of p62 at the transcriptional level, through increased synthesis of the protein.

p62 turnover by trehalose was determined to evaluate the effect of trehalose on p62 synthesis. The basal level of p62 synthesis in RPE cells was determined by incubating cells with chloroquine (CQ) to inhibit autophagy and abolish p62 degradation. The p62 protein level was compared in cells incubated for 24 h with 50 *μ*M CQ alone to cells coincubated with 50 mM trehalose in the presence of 50 *μ*M CQ. Densitometric analysis of our western blot showed that cells coincubated with trehalose in the presence of CQ expressed higher p62 levels compared to those incubated with only CQ or trehalose (Figures 2(b) and 2(d)). These results support an increase in the synthesis in p62 by trehalose, as lysosomal degradation was abolished in both samples.

### 3.4. Trehalose Enhanced Proteasome Degradation Involved in Downregulating p62 Protein Levels

**Figure 3:**
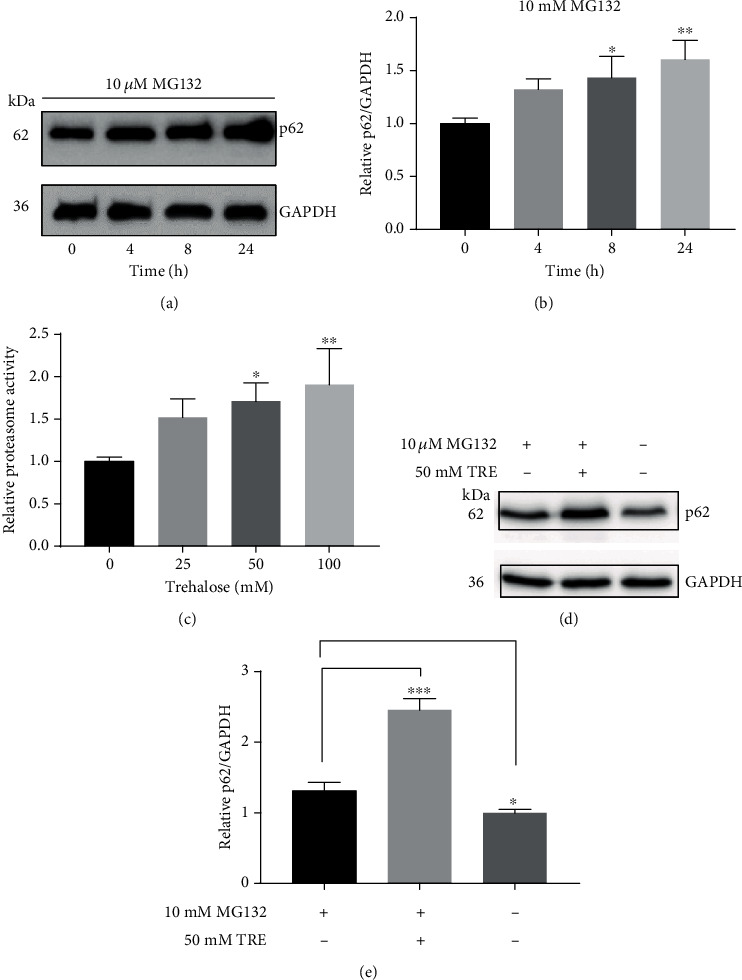
Accumulation of p62 by trehalose was not due to impaired proteasome degradation. (a, b) Time-dependent increase in p62 protein level by MG132 in cell lysate using immunoblot. (c) Proteasome activity level in whole-cell lysates from cells. (d) Trehalose increased p62 level in the presence of MG132. Data represent the mean ± SD of 3 independent experiments. Statistical analysis was performed by one-way ANOVA followed by multiple comparison tests. ^∗^*p* < 0.05, ^∗∗^*p* < 0.001 vs. control.

The UPS also plays an important role in proteolysis and may be involved in regulating the p62 level in RPE cells. To determine the contribution of the UPS to the p62 level, cells were incubated with the proteasome inhibitor MG132. Inhibition of proteasomal degradation by MG132 promoted the accumulation of p62 in the RPE cells (Figures 3(a) and 3(b)), indicating the regulatory role of UPS. On this premise, the proteasome activity level of cells incubated with trehalose was evaluated to determine whether the disaccharide impaired proteasome degradation and elevated p62 levels. On the contrary, trehalose significantly increased proteasome activity in the cells, indicating that impaired proteasome degradation had no role in the accumulation of p62 (Figure 3(c)). Furthermore, cells were coincubated with trehalose and MG132 to investigate the possible mechanism responsible for the increase in p62 protein level by trehalose. In the presence of the proteasome inhibitor MG132, trehalose still increased the p62 level (Figures 3(d) and 3(e)). Collectively, the evidence points to increased synthesis of p62 as the cause of the upregulation of p62 expression rather than of inhibition of proteolysis by trehalose.

### 3.5. Trehalose Promotes Chaperone-Mediated Autophagy and Lysosomal Degradation

**Figure 4:**
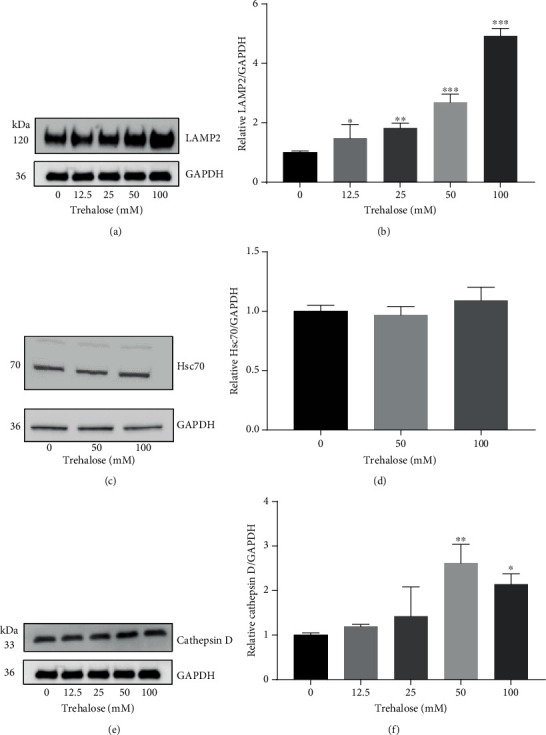
Effect of trehalose on LAMP2, Hsc 70, and cathepsin D protein levels in ARPE-19 cells. (a–f) Immunoblotting with appropriate antibodies in whole-cell lysate from trehalose-treated cells. Data represent the mean ± SD of 3 independent experiments. Statistical analysis was performed by one-way ANOVA followed by multiple comparison tests. ^∗^*p* < 0.05, ^∗∗^*p* < 0.01, ^∗∗∗^*p* < 0.001 vs. control.

*LAMP2* is a lysosomal marker that regulates the formation of autolysosomes, as it promotes the fusion of autophagosomes to lysosomes [46]. The LAMP2 expression level is the limiting factor in the degradation of substrates via CMA, a selective lysosomal degradative pathway that depends on LAMP-2A (Susmita [47]). Therefore, the effect of trehalose on autolysosome formation, as well as CMA, was investigated by examining the LAMP2 expression changes by western blot. The results showed that LAMP2 expression was upregulated dose-dependently in cells incubated with trehalose (Figures 4(a) and 4(b)), providing evidence for an increase in autolysosome formation and CMA in the presence of the disaccharide.

Hsc 70 is another essential molecular chaperone involved in the regulation of CMA, by facilitating the specific recognition of CMA substrates in the cytosol for lysosomal uptake (Susmita [47]). The binding of Hsc70 to the KFEFQ-motif is sufficient and necessary for the lysosomal uptake of a substrate for degradation (Susmita [47]). It was, therefore, determined whether any changes occurred in the level of this constitutively expressed protein by trehalose, such as found when treated with the autophagy inhibitor and neurotoxin rotenone [48]. Interestingly, trehalose did not affect or dysregulate Hsc 70 protein expression (Figures 4(c) and 4(d)).

To promote autolysosome formation necessitated the upregulation of lysosomal degradation [49], the final stage in autophagy, which is completed by the hydrolytic enzymes within the lysosomal lumen. Among the several hydrolases, cathepsins are noted for the degradation of a wide range of autophagy substrates [50]. Inhibition of cathepsin B and D was found to impair autolysosome degradation in fibroblasts [51]. Hence, the effect of trehalose on cathepsin D protein expression in RPE cells was determined. The results demonstrated that trehalose upregulated the expression of cathepsin D dose-dependently (Figures 4(e) and 4(f)), indicating an enhancement of degradation by lysosomes. These results also support the promotion of autolysosome formation and CMA by trehalose in human RPE cells.

### 3.6. Trehalose Upregulates LAMP2 via a Macroautophagy-Independent Mechanism

**Figure 5:**
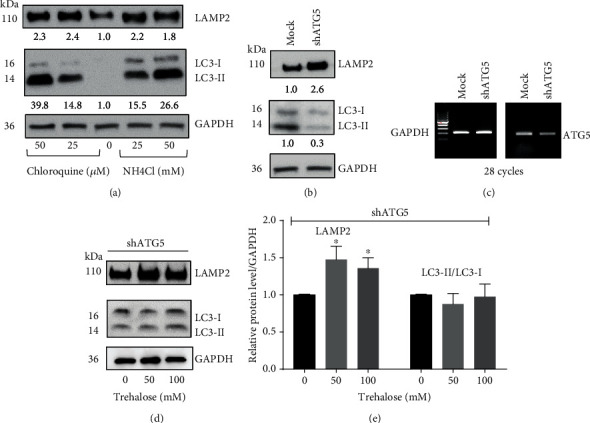
Trehalose promotes LAMP2 expression independent of macroautophagy in human RPE cells. (a, b) Immunoblots showing an upregulation of LAMP2 expression by autophagy inhibitors chloroquine (CQ) and NH4Cl, and shRNA ATG5 knockdown. (c) RT-PCR evaluation of ATG5 knockdown. (d) Immunoblots showing increased LAMP2 in ATG5 knockdown cells without affecting macroautophagy. Data represent the mean ± SD of 3 independent experiments. Statistical analysis was performed by one-way ANOVA followed by multiple comparison tests. ^∗∗^*p* < 0.01, ^∗∗∗^*p* < 0.001 vs. control.

Under certain conditions of stress, CMA may be upregulated to compensate for macroautophagy inhibition (S. [52]). The existence of this kind of “crosstalk” between the two pathways in the RPE cells was validated by inhibiting macroautophagy with two lysosomotropic agents (CQ and NH_4_Cl) or transfection of cells with shRNA ATG5. The data showed inhibition of macroautophagy with CQ or NH_4_Cl (evidenced by the accumulation of LC3-II) upregulated the LAMP2 protein expression dose-dependently (Figure 5(a)), supporting CMA upregulation to compensate for impaired macroautophagy. Similarly, LAMP2 was highly expressed in shATG5-knockdown cells compared to wildtype cells, indicating increased CMA activity (Figure 5(b)). Autophagy inhibition in shATG knockdown cells was validated by RT-PCR for ATG5 mRNA level (Figure 5(c)) and western blot for LC3-II level (Figure 5(b)). To demonstrate that trehalose upregulates CMA independent of macroautophagy, shATG5 cells were incubated with different doses of trehalose for 24 h and examined the LAMP2 expression levels. Interestingly, trehalose increased LAMP2 in ATG5 knockdown autophagy-defective cells without any change in LC3-II (Figures 5(d) and 5(e)). These data demonstrated that LAMP2 upregulation by trehalose was independent of macroautophagy.

### 3.7. Transcriptional Upregulation of TFEB and Autophagy by Trehalose

**Figure 6:**
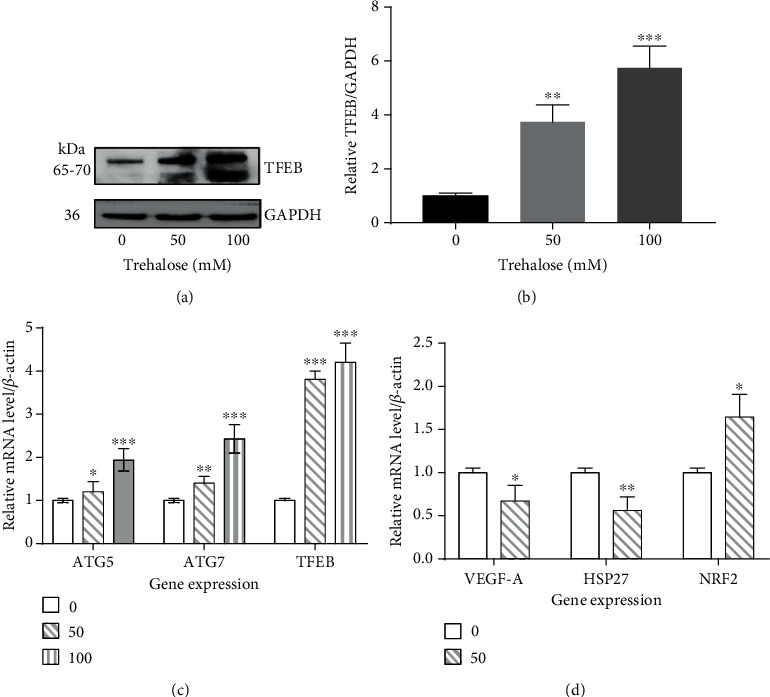
Changes in the mRNA and/or protein levels in the autophagy-lysosomal pathway and oxidative stress. (a, b) Immunoblot showing an increase in the expression of TFEB in whole-cell lysate by trehalose. (c, d) RT-qPCR showing transcriptional changes in autophagy genes ATG5 and ATG7 and oxidative stress pathway targets, including NRF2, HSP 27, and VEGF-A. Data represent the mean ± SD of 3 independent experiments. Statistical analysis was performed using either unpaired *t* test or one-way ANOVA test, followed by Dunnett's post hoc test; ^∗^*p* < 0.05, ^∗∗^*p* < 0.01, ^∗∗∗^*p* < 0.001.

TFEB plays a pivotal role in the regulation of autophagy [17, 53]. TFEB overexpression leads to the activation and nuclear translocation of TFEB, resulting in the upregulation of lysosomal genes and autophagy [17, 53]. Also, TFEB activation has a positive feedback on its own transcriptional expression, which is evident in starvation where TFEB activation and nuclear translocation leads to the upregulation of TFEB mRNA levels in mice [53]. Hence, increased TFEB mRNA and protein expression is an indication of TFEB activation and autophagy. This investigation showed that TFEB was upregulated at the transcriptional and protein levels in the trehalose-treated cells compared to control (Figures 6(a)–6(c)). In addition, the mRNA levels of ATG5 and ATG7 were increased in the trehalose-treated cells compared to control (Figure 6(c)). These findings reveal transcriptional regulation of TFEB and autophagy by trehalose, corroborating the results of our earlier work and that of other researchers [22, 23].

### 3.8. Transcriptional Regulation of NRF2, Hsp27, and VEGF-A

The effects of trehalose on the gene expression levels of key protein molecules implicated in or linked to oxidative stress and AMD were explored. These included the vascular endothelial growth factor-A (*VEGF-A*), heat shock protein 27 *(HSP27)*, and transcription factor nuclear factor erythroid 2-related factor 2 gene (*NFE2L2/NRF2)* [7, 54, 55]. In the retina or RPE of AMD donor eyes, the protein or mRNA levels of *VEGF-A* and *HSP27* are upregulated, while NRF2 is inhibited [10, 56, 57]. The upregulation of individual VEGF-A isoforms disrupts retinal homeostasis, inciting the onset and progression of neovascular AMD [57]. As NRF2 activation protects against oxidative stress, its impairment promotes oxidative damage to RPE, an insult that drives AMD. Hsp27 is a molecular chaperone that is reported to regulate misfolding of proteins, actin reorganization, and key components of the apoptotic signaling pathway in the retina [55, 58, 59], but its exact role, whether protective or harmful, is controversial. It was reported to contribute to RPE membrane blebbing and sub-RPE deposits in mice exposed to the cigarette smoke oxidant HQ [55]. Cells treated with trehalose downregulated the mRNA levels of *VEGF-A* and *Hsp27* and upregulated the *NRF2* antioxidant transcription factor relative to control (Figure 6(d)). These results suggested that trehalose may modulate oxidative stress in human RPE cells.

### 3.9. Autophagy Induction by Trehalose Was Not Associated with Oxidative Stress or Cell Death

**Figure 7:**
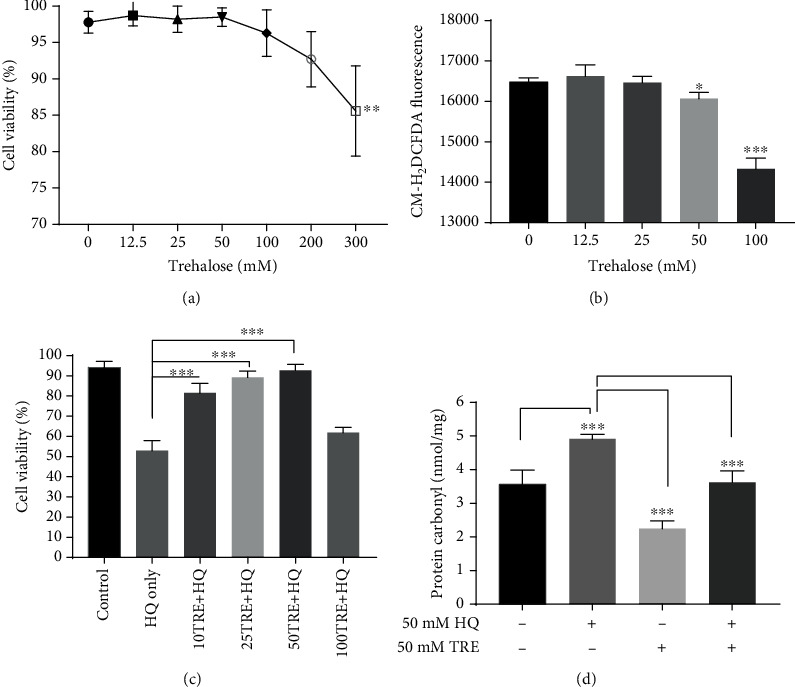
Cytoprotection of trehalose against oxidative damage by hydroquinone (HQ) in ARPE-19 cells. (a) Trypan blue assay reveals that trehalose is nontoxic over a wide dose range. (b) Trehalose decreased basal level of reactive oxygen species (ROS) assessed using CM-H_2_DCFDA assay (c, d) Trehalose pretreatment improves the viability of cells and lowered the protein carbonyl levels in cells exposed to HQ. Data represent the mean ± SD of 3 independent experiments. Statistical analysis was performed by one-way ANOVA test, followed by multiple comparison *post hoc* test; ^∗^*p* < 0.05, ^∗∗∗^*p* < 0.001.

Under conditions of stress or cell death, autophagy is upregulated for cytoprotection [60]. Hence, investigating whether an autophagy inducer increased reactive oxygen species (ROS) production and its cytotoxicity is relevant to inform on the therapeutic role of that agent. Trypan blue dye exclusion assay revealed no changes in the viability of human RPE cells incubated with doses of trehalose up to 200 mM for 24 h (Figure 7(a)), indicating that trehalose has a wide nontoxic dose range for harnessing its autophagy effect. Additionally, the CM-H2DCFDA assay for intracellular ROS measurement showed that the cells incubated with trehalose for 24 h had significantly lower ROS levels compared to control (Figure 7(b)). Altogether, the results showed that autophagy induction by trehalose was independent of oxidative stress or toxicity, as the disaccharide rather possessed antioxidant properties.

### 3.10. Trehalose Protects against Oxidative Damage from the Oxidant Hydroquinone (HQ)

Mice fed with the cigarette smoke oxidant HQ developed subretinal deposits, oxidative damage of the RPE, and retinal degeneration [61, 62]. These findings implicated HQ as a risk factor for developing AMD in cigarette smokers. The cytoprotective effect of trehalose against HQ-induced toxicity in RPE cells was evaluated using assays for protein carbonyl and cell viability. Protein carbonyl is a stable and reliable marker that directly correlates with oxidative stress levels [63]. When cells were exposed to 50 *μ*M HQ for 2 h without trehalose pretreatment, cells showed significantly lower viability and higher protein carbonyl levels compared to the control (*p* < 0.001, Figures 7(c) and 7(d)), indicating increased oxidative damage. However, pretreatment of cells with trehalose for 24 h prevented the loss of cell viability and led to an increase in the protein carbonyls level in a dose-dependent manner (Figures 7(c) and 7(d)). Strangely, it was found that doses of trehalose of 100 mM trehalose or above were not protective, despite their potent-ROS inhibition (Figure 7(c)). This observation also contributed to the use of a lower dose of trehalose (50 mM) throughout the experiments. It is possible that the marked reduction in ROS levels by higher doses of trehalose disrupts redox-signaling pathways. While ROS were regarded as harmful, it is emerging that they may be relevant as signal transducers, and are involved in the regulation of several cellular processes [64]. Perhaps, also the upregulation of autophagy contributed to the loss of viability, as beyond certain limits autophagy could trigger apoptosis [65].

### 3.11. Trehalose's Cytoprotection against HQ-Induced Oxidative Damage Was Dependent on Autophagy Induction

**Figure 8:**
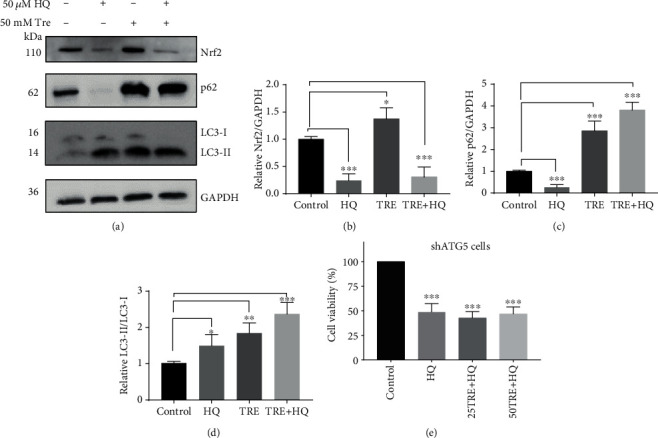
Autophagy upregulation by trehalose protects against hydroquinone- (HQ-) induced oxidative stress in human RPE-19 cells. (a–d) Immunoblots for changes in protein expression of LC3-II, p62, and Nrf2 in cells preincubated with trehalose or vehicle before HQ exposure. (e) Trypan blue assay showing loss of protection against HQ in shRNA ATG5 cells pretreated with trehalose. Data represent the mean ± SD of 3 independent experiments. Statistical analysis was performed by one-way ANOVA test, followed by Dunnett's *post hoc* test; ^∗^*p* < 0.05, ^∗∗^*p* < 0.01, ^∗∗∗^*p* < 0.001.

To elucidate the mechanisms behind the cytoprotection of trehalose against HQ, we studied differences in the protein expression of LC3-II, p62, and NRF2 in cells treated with 50 mM trehalose or vehicle before incubation with HQ. Cells incubated with 50 *μ*M HQ marginally increased LC3II (*p* = 0.04) and decreased p62 and NRF2 protein levels (Figures 8(a)–8(c), *p* < 0.001), suggesting impairment of the NRF2 antioxidant pathway while autophagy was activated [66]. Trehalose treatment upregulated the expression of LC3II, p62, and NRF2 in cells, supporting the activation of both autophagy and NRF2 pathways (Figures 8(a)–8(c)). Trehalose pretreatment, however, did not prevent the depletion of NRF2 in cells following HQ exposure (Figures 8(a) and 8(b)), but the protein levels of LC3II and p62 were upregulated (Figures 8(c) and 8(d), *p* < 0.001). These results linked the cytoprotection of trehalose to the upregulation of autophagy and/or p62. To confirm the role of autophagy activation, the cytoprotection of trehalose-pretreatment against HQ in shRNA ATG5 cells was also explored. The viability of shRNA ATG5 cells pretreated with trehalose was lowered to a level similar to that in the untreated cells (Figure 8(e)), demonstrating the loss of cytoprotection by trehalose in autophagy impaired cells. Our data, therefore, support autophagy activation by trehalose in RPE cells to protect against HQ.

## 4. Discussion

The retinal pigment epithelium (RPE) is an essential monolayer of pigmented cells of the retina, whose functions include the recycling of rhodopsin chromophores, nourishment of photoreceptors, phagocytosis of the photoreceptor outer segment, and formation of the retinal-blood barrier [67, 68]. The RPE cells in healthy adult eyes, similar to other neurons in the human body, are postmitotic in situ [69], and, hence, maintenance of RPE homeostasis is crucial for effective retinal function. In AMD and some other retinal degenerative conditions, the RPE is the earliest targeted site of damage, particularly by oxidative stress [67]. Therefore, interventions protective against oxidative damage in the RPE may have therapeutic relevance in the management of retinal diseases. Data from this study showed that autophagy induction by trehalose effectively protected human RPE cells against HQ-induced oxidative stress. This has important clinical implications, since finding a treatment modality that inhibits oxidative damage in human RPE cells by cigarette smoke oxidants could potentially prevent the development of AMD. Cigarette smoking, an important risk factor in AMD, causes oxidative damage to the RPE and HQ in smoke/tar was implicated as a major oxidizing agent [6, 62, 70].

The current study investigated the autophagy-stimulating effect of trehalose. Since autophagy is dynamic, both autophagy specific markers and autophagy flux were examined to determine the effect of trehalose on each stage of the autophagy-lysosome pathway. The autophagy markers and autophagy flux were upregulated by the disaccharide in the human RPE cell line. TFEB overexpression and autophagy upregulation by trehalose found in this study are consistent with recent studies investigating the effect of trehalose *in vivo* in mice and primary cells [22, 23]. Thus, the autophagy-inducing effect of trehalose is not limited by cell type.

Previous studies and our current study found that trehalose increased p62, which could be suggestive of autophagy inhibition [44]. The accumulation of p62 by trehalose, theoretically, could occur under two conditions; (1) inhibition of lysosomal degradation and (2) upregulation of p62 synthesis. This study demonstrated that trehalose upregulated LAMP-2 and cathepsin D, indicators of enhanced autophagosome-lysosome fusion and lysosomal degradation in cells, respectively (Susmita [47, 51]). In addition, it was shown that the accumulation of p62 was correlated with transcriptional upregulation of the p62 gene, explaining the possibility of increased p62 protein synthesis by trehalose. It is important to note that p62 accumulation under autophagy is not an isolated case for trehalose, as upregulation of p62 at the transcription level has been observed in starvation-induced autophagy [45]. The finding of potent autophagy induction in human RPE cells by trehalose will be of great interest because of the recent insight into the role of autophagy in AMD and other retinal degenerative diseases [71, 72].

The results showcase trehalose as a unique autophagy inducer due to its dual stimulation of macroautophagy and CMA, and protection against oxidative stress associated with NRF2 inhibition in human RPE cells. The cytoprotective role of autophagy against oxidative stress is being studied to facilitate the control of this process. Macroautophagy and CMA perform different lysosomal degradation roles, with the latter being more selective compared to the former [47]. Although the two mechanisms may compensate for each other when there is a compromise in one, by way of upregulating the functional autophagy pathway, the activation of both autophagy pathways is needed for effective cellular resistance against oxidative stress [73]. While this study directly evaluated the role of macroautophagy, the involvement of CMA could not be overlooked as trehalose efficiently upregulated LAMP2. The upregulation of the CMA receptor LAMP-2A in breast cancer cells was found to protect cells from oxidative damage and reduce oxidative modification of cellular proteins [74]. It is, therefore, clear that the dual stimulation of both macroautophagy and CMA may be the reason for the efficacy of trehalose in dealing with oxidative stress irrespective of the impairment of NRF2 in RPE cells.

While NRF2 activation directly inhibits the accumulation of ROS, autophagy ameliorates oxidative damage by being involved in the repair of damage caused by ROS-induced cell injury. This occurs by the removal of damaged cytosolic molecules and organelles, including proteins, lipids, and damaged mitochondria, and recycling the nutrients for synthesis of new molecules and organelles [75]. For instance, autophagy has been shown to aid in DNA damage response through its regulation of p62 levels that could otherwise inhibit the DNA repair process [76]. Thus, while the role of autophagy in maintaining protein homeostasis, known as proteostasis, is essential under normal conditions [71], it is even more beneficial under oxidative stress due to impairment of the proteasome degradation that accompanies oxidative stress in the RPE [77]. Upregulating autophagy can alleviate the burden in the RPE for proteolytic degradation, which is higher under oxidative stress and, therefore, may help restore proteostasis.

The impairment of NRF2 under oxidative stress-related conditions such as aging, cigarette smoking, and AMD has been reported [10–12]. It was found that trehalose also promotes NRF2 expression, evident by the increase of NRF2 transcription factor and protein levels and reduction of basal ROS levels in the treated cells. However, NRF2 activation by trehalose did not occur in the presence of the cigarette oxidant HQ. This finding is worth noting as it suggests that strategies to inhibit oxidative stress through the activation of the NRF2 antioxidant pathway might be ineffective. This suggests the importance of targeting the autophagy pathway in RPE cells under oxidative stress conditions. The dysregulation of the autophagy mechanism was found to be associated with susceptibility to oxidative stress and AMD [16], an indication that autophagy promotes cell survival under oxidative stress. Although trehalose showed cytoprotection against HQ, at higher concentration of trehalose was not protective against HQ. This may be related to autophagy-induced cell death occurring due to overactivation, as has been reported in some conditions [78–80]. This finding calls for exercising caution on the dose of trehalose to be used if cytoprotection against oxidative damage is desired.

The cytoprotection of trehalose against oxidative stress may be mediated by diverse biomolecular signaling pathways, including autophagy, NRF2 overexpression, molecular chaperone Hsp 27, and VEGF, as reported here and in other studies [21, 29]. This study, however, delineated autophagy induction by trehalose as the cytoprotective mechanism against HQ-induced toxicity, by showing that NRF2 depletion still occurred in trehalose-pretreated cells protected from HQ damage. Furthermore, it was shown that the inhibition of autophagy, through shATG5 knockdown, resulted in almost complete loss of the protection of trehalose against HQ. Due to the vital role of NRF2 in the transcriptional regulation of antioxidant genes and enzymes to reduce ROS levels, the cytoprotection of trehalose against HQ-induced toxicity reveals autophagy as a powerful redox signaling regulator capable of compensating for oxidative stress associated with dysregulation of NRF2. Extra measures were undertaken to eliminate potential confounders in the cytoprotection against HQ, due to a possible redox reaction could occur between trehalose and HQ. To avoid this, cells were washed three times to remove trehalose before incubation with HQ.

The autophagy machinery functions as an integrated cellular stress response, dictating the fate of cells—whether cells survive or die—depending on factors including the stress level [9, 79]. It was found that when cells were incubated with higher doses of trehalose, its cytoprotection against HQ-induced toxicity was diminished or lost, although higher doses of trehalose induced greater levels of autophagy and higher ROS inhibition. The loss of protection may be due to overstimulation of autophagy resulting in apoptotic death of cells, as occurs under certain conditions such as prolonged starvation and oxidative stress [65, 81, 82], since the trehalose-treated cells showed greater LC3-II levels following exposure to HQ (Figure 8(d)). Thus, there may be a limit for autophagy induction to be protective in cells, which indicates a need for further studies on what triggers the switch from a survival to a suicidal mission.

In conclusion, this study validates trehalose as a potent TFEB-mediated autophagy inducer in human RPE cells that is cytoprotective in an *in vitro* AMD condition mimicked using hydroquinone. Studies to ascertain the neuroprotection of trehalose in alleviating animal models of AMD and retinal diseases associated with impaired NRF2 antioxidant defense are, therefore, warranted.

## Figures and Tables

**Table 1:** List of primers used for RT-PCR and qPCR.

Gene	Primer sequence
Human *β*-actin	Forward primer: 5′-CCAACCGCGAGAAGATGA-3′ Reverse primer: 5′-CCAGAGGCGTACAGGGATAG-3′

Human TFEB	Forward primer: 5′-CCAGAAGCGAGAGCTCACAGAT-3′ Reverse primer: 5′-TGTGATTGTCTTTCTTCTGCCG-3′

Human ATG5	Forward primer: 5′-AAGCTGTTTCGTCCTGTGGC-3′ Reverse primer: 5′-CCGGGTAGCTCAGATGTTCA-3′

Human ATG7	Forward primer: 5′-CGTTGCCCACAGCATCATCTTC-3′ Reverse primer 5′-TCCCATGCCTCCTTTCTGGTTC-3′

Human NRF2	Forward primer: 5′-ACACGGTCCACAGCTCATC-3′ Reverse primer: 5′-TGTCAATCAAATCCATGTCCTG-3′

Human p62	Forward primer: 5′-TGCCCAGACTACGACTTGTG-3′ Reverse primer: 5′-AGTGTCCGTGTTTCACCTTCC-3′

Human VEGF-A	Forward primer: 5′-TGCCATCCAATCGAGACCCTG-3′ Reverse primer: 5′-GGTGATGTTGGACTCCTCAGTG-3′

Human Hsp27	Forward primer: 5′-TCCCTGGATGTCAACCACTT-3′ Reverse primer: 5′-GATGTAGCCATGCTCGTCCT-3′

## Data Availability

All data reported in this study is available upon request from the authors.
